# Aortic Distensibility Measured by Automated Analysis of Magnetic Resonance Imaging Predicts Adverse Cardiovascular Events in UK Biobank

**DOI:** 10.1161/JAHA.122.026361

**Published:** 2022-12-06

**Authors:** Marina Cecelja, Bram Ruijsink, Esther Puyol‐Antón, Ye Li, Harriet Godwin, Andrew P. King, Reza Razavi, Phil Chowienczyk

**Affiliations:** ^1^ King’s College London British Heart Foundation Centre, Department of Clinical Pharmacology St Thomas’ Hospital London United Kingdom; ^2^ School of Bioengineering and Imaging Sciences King’s College London London United Kingdom; ^3^ King’s College London British Heart Foundation Centre School of Cardiovascular Medicine & Sciences, Department of Cardiology London United Kingdom

**Keywords:** aortic stiffness, distensibility, outcome, Cardiovascular Disease

## Abstract

**Background:**

Automated analysis of cardiovascular magnetic resonance images provides the potential to assess aortic distensibility in large populations. The aim of this study was to compare the prediction of cardiovascular events by automated cardiovascular magnetic resonance with those of other simple measures of aortic stiffness suitable for population screening.

**Methods and Results:**

Aortic distensibility was measured from automated segmentation of aortic cine cardiovascular magnetic resonance using artificial intelligence in 8435 participants. The associations of distensibility, brachial pulse pressure, and stiffness index (obtained by finger photoplethysmography) with conventional risk factors was examined by multivariable regression and incident cardiovascular events by Cox proportional‐hazards regression. Mean (±SD) distensibility values for men and women were 1.77±1.15 and 2.10±1.45 (*P*<0.0001) 10^−3^ mm Hg^−1^, respectively. There was a good correlation between automatically and manually obtained systolic and diastolic aortic areas (*r*=0.980 and *r*=0.985, respectively). In regression analysis, distensibility associated with age, mean arterial pressure, heart rate, weight, and plasma glucose but not male sex, cholesterol or current smoking. During an average follow‐up of 2.8±1.3 years, 86 participants experienced cardiovascular events 6 of whom died. Higher distensibility was associated with reduced risk of cardiovascular events (adjusted hazard ratio [HR], 0.61 per log unit of distensibility; *P*=0.016). There was no evidence of an association between pulse pressure (adjusted HR 1.00; *P*=0.715) or stiffness index (adjusted HR, 1.02; *P*=0.535) and risk of cardiovascular events.

**Conclusions:**

Automated cardiovascular magnetic resonance‐derived aortic distensibility may be incorporated into routine clinical imaging. It shows a similar association to cardiovascular risk factors as other measures of arterial stiffness and predicts new‐onset cardiovascular events, making it a useful tool for the measurement of vascular aging and associated cardiovascular risk.

**Nonstandard Abbreviations and Acronyms**
PP: pulse pressure
SI: arterial stiffness index

**Clinical Perspective**

**What Is New?**

- This is the largest study to date to demonstrate the ability of aortic distensibility as determined by automated cardiovascular magnetic resonance to significantly predict new‐onset cardiovascular events.

**What Are the Clinical Implications?**

- With cardiovascular magnetic resonanceimaging increasingly being used in a clinical setting, measures of aortic stiffness by cardiovascular magnetic resonanceare likely to become more common.

Vascular aging is characterized by a widening of pulse pressure (PP) with isolated systolic hypertension, the most frequent subtype of hypertension seen in older individuals.^1^ An important determinant of PP is decreased distensibility of the aorta,^2^, ^3^, ^4^ which limits its buffering capacity to accommodate blood ejected from the left ventricle. Distensibility is a direct local measure of aortic stiffness, calculated as the relative change in aortic diameter divided by the local aortic PP (often estimated from brachial PP).^5^ Aortic distensibility can be accurately measured by cardiovascular magnetic resonance imaging (CMR). When measured in the ascending aorta^6^, ^7^ and aortic arch,^8^ by CMR, aortic distensibility predicts adverse cardiovascular outcomes in individuals without overt cardiovascular disease. PP obtained simply from brachial blood pressure (BP) is often used as a surrogate marker of arterial stiffness, although it also influenced by left ventricular ejection properties and aortic anatomy. Arterial stiffness index (SI) derived from finger photoplethysmography is another surrogate measure of arterial stiffness, which correlates with pulse wave velocity (PWV; regarded as the “gold‐standard” measure of arterial stiffness).^9^ We sought to investigate the relative associations of CMR‐derived aortic distensibility, PP, and SI with cardiovascular risk factors and cardiovascular events in participants from UK Biobank, a large community dwelling cohort. We derived distensibility automatically through segmentation of aortic cine CMR using quality‐controlled artificial intelligence. With CMR being used increasingly in clinical settings such a measure of distensibility could be derived during routine imaging with no additional overhead and applied for risk stratification in clinical practice with similar ease to the PP and SI.

## METHODS

### Data Access

The data used in this study are available to all bona fide researchers via application to the UK Biobank in accordance with their approval criteria. Information for the detailed access procedure can be found at https://www.ukbiobank.ac.uk/.

### Participants

This research has been conducted using the UK Biobank Resource under Application Number 55014. UK Biobank is a community‐based prospective population study of >500 000 participants aged between 40 and 69 conducted in the United Kingdom and has previously been described in detail.^10^ Baseline data were collected between 2006 and 2010. The present study was performed in a subsample of 12 491 apparently healthy individuals who took part in the third imaging visit (Instance 2) from 2014 and who underwent magnetic resonance imaging (MRI). Subjects with previously diagnosed cardiovascular disease or clinically significant disease impacting cardiovascular physiology (see full exclusion criteria in^11^) were excluded from the study.

Cardiovascular risk factors considered included sex, BP, height and weight, smoking, and glucose.^12^, ^13^, ^14^ Age, medical history, medication, height, weight, and smoking status were recorded during the imaging visit. Smoking status was coded as 1 if current smoker and 0 if not a current smoker. Heart rate and brachial systolic and diastolic BP were measured during the same imaging visit using an automated device (Omron 705, OMRON Healthcare Europe B.V. Kruisweg 5 772 132 NA Hoofddorp). An appropriate BP cuff was selected based on the measured circumference of the midpoint of the upper arm. Two consecutive measurements were taken sequentially on the left upper arm by a registered nurse with the participant quietly seated and with no restrictive clothing to the left arm. Mean arterial pressure (MAP) was calculated as [(2×diastolic BP)+systolic BP]/3. PP was defined as brachial systolic BP − diastolic BP. SI was measured during the imaging visit using the PulseTrace PCA2 device (CareFusion, San Diego, CA), which uses finger photoplethysmography to obtain pulse waveforms as previously described.^15^ In brief, pulse waveforms were obtained by placing an infrared light emitting diode and sensor on the participants finger or thumb, preferably the index finger of the nondominant arm, and recording a photoplethysmograph pulse waveform over 10 to 15 seconds with the participant supine. SI in m/sec was calculated by dividing participant height by time between the peaks of the pulse waveform, which represents an estimate of the time taken for components of the pulse waves to travel to peripheral sites and be reflected back. Ethnicity, cholesterol (including low‐density lipoprotein cholesterol and high‐density lipoprotein [HDL] cholesterol), and glucose were recorded during the baseline visit. In cases where variables other than aortic diameters were not available at the imaging visit, they were taken from the baseline (or in a few cases from the first) visit (n=357 for weight, n=66 for diabetes, n=56 for smoking status, n=2283 for BP, n=136 for total cholesterol, n=229 for HDL cholesterol, and n=231 for glucose). UK Biobank received approval for the study from the National Information Governance Board for Health and Social Care and the National Health Service North West Centre for Research Ethics Committee (Ref: 11/NW/0382) and written consent was obtained from all participants. The UK Biobank consent form is available at http://www.ukbiobank.ac.uk/wp‐content/uploads/2011/06/Consent_form.pdf.

### MRI Imaging and Aortic Distensibility

MRI imaging was conducted during the third visit from April 30, 2014 to March 2020 using a Siemens 1.5 Tesla MAGNETOM Aera scanner (Siemens Healthineers, Erlangen, Germany) as previously described.^16^ Retrospective ECG‐gated cine images were acquired using a transverse cine balanced Steady State Free Precession sequence at the level of the pulmonary trunk and right pulmonary artery during breath‐hold. Typical acquisition parameters were echo time=1.17 ms, repetition time=2.8 ms, flip angle=80°, Grappa factor=2, acquired matrix size=240 × 196, voxel size=1.6 × 1.6 × 6 mm^3^, and actual temporal resolution=28 ms, which was interpolated to 100 images per cardiac cycle with resolution ≈10 ms. For the automatic analysis, ground truth data were manually generated by a team of 2 observers under the guidance of 2 principal investigators.

### Automatic Image Analysis

The proposed framework for automatically quantifying aortic distensibility from cine MR sequences consists of (1) an image segmentation network that automatically segments the ascending aorta, and (2) a quality control (QC) network that checks the change in aortic area over time to detect abnormalities in the aortic area‐time curve over the cardiac cycle.

#### Image Segmentation Network

For automated segmentation, we used a fully convolutional network with a 17 convolutional layer Visual Geometry Group‐like architecture. From the output of the network, we can compute the cross‐section area of the ascending aorta during a cardiac cycle and select the minimum (Amin) and maximum (Amax) areas to compute distensibility as (Amax–Amin)/(Amin×PP) in mm Hg^−1^.^6^ The network was trained using ground truth segmentations of cine CMR images in 200 subjects, consisting of both healthy volunteers as well as patients with a variety of cardiac diseases (to provide as wide a range of physiology as possible). For each subject, 5 random frames of the cardiac cycle were manually segmented. During training we used an 80/20 training/validation split to monitor the performance of the network. A further test database of 100 subjects (50 healthy subjects and 50 subjects with cardiac disease) where all frames of the cardiac cycle were manually segmented was used to validate the segmentation network (see Statistical Analysis). Data augmentation was performed using random translation, rotations, scaling, and intensity transformations.

#### Quality Control

The segmentation network outputs a cross‐section area of the aorta for each frame of the cardiac cycle. Quality of image acquisition is of particular importance when measuring distensibility because errors in quantification of distensibility can arise from a range of different image acquisition or processing quality issues, including tortuosity of the aorta, planning of the acquisition plane, arrythmias, and breathing artifact. All these quality issues result in changes in the characteristic of the aortic area change over time. We implemented a convolutional neural network long short‐term memory network^17^ to interrogate the change in area over the cardiac cycle and detect unphysiological and erroneous change in aortic area over the cardiac cycle. The developed convolutional neural network long short‐term memory network combines a convolutional neural network model for feature extraction and the long short‐term memorymodel for interpreting the features across time steps. The convolutional neural network long short‐term memory network assessed resembled a physiological waveform. An experienced CMR cardiologist, blinded to the automated pipeline's verdict, critically reviewed the segmentations and curves of aortic cross‐section area over time and classified them as correct or erroneous based on the waveform morphology. Our developed QC step detects these changes and is therefore agnostic of the underlying errors. The training database for this process consisted of 1200 subjects, both healthy subjects and subjects with a cardiac disease. During training we used an 80/20 training/validation split to monitor the performance of the network. A further test database of 300 subjects (including healthy subjects and those with cardiac disease) was as used to validate the QC network (see Statistical Analysis).

Out of the 12 491 participants with MRI images available for analysis, aortic area was rejected by the QC steps in 3775 participants. Distensibility was calculated using the automated method in the remaining 8716 patients. BP was missing in a further 281 individuals leaving 8435 individuals with distensibility measurements (Table S1–S4).

### Ascertainment of Cardiovascular Death

Participant follow‐up for mortality was censored on May 2020. Cause of death was obtained from the National Health Service Information Centre as detailed online at http://biobank.ctsu.ox.ac.uk/crstal/refer.cgi?id=115559. The primary cause of death was used to identify cardiovascular death coded according to the *International Classification of Diseases, 10th Revision (ICD‐10)* ^17^ as myocardial infarction (I21‐23, I252), coronary heart disease (I21‐25, Z951, Z955), heart failure (I110, I130, I132, I50), and stroke (I60, I61, I629, I63, I64, I678, I690, I693, G951, H341, H342, S066).^15^

### Ascertainment of Cardiovascular Events

Incident cardiovascular events (myocardial infarction, coronary heart disease, heart failure, and stroke) were recorded until March 2020. All UK Biobank participants have been linked to their hospital inpatient data, which are provided by National Health Service Digital in England, Information and Statistics Division in Scotland, and Secure Anonymised Information Linkage in Wales. Primary diagnosis of a cardiovascular event was made during hospitalization and coded using *ICD‐10* as described. In addition, new diagnoses of angina pectoris (I20.0, I20.9), dilated cardiomyopathy (I42.0), peripheral vascular disease (I73.9), and occlusion or stenosis of a carotid artery (I65.2) were included as cardiovascular events. Date of event was defined as the date of first in‐patient diagnosis.

### Statistical Analysis

Data are expressed as mean± SD for continuous variables or as a count and percentage for categorical variables. Three participants had improbable distensibility values and were excluded from further analysis, leaving 8435 participants with distensibility measures. Baseline participant characteristics were compared in those with (n=8435) and without distensibility measurements (n=3778) and in the total UK Biobank cohort. Log transformations were applied to glucose and distensibility values to obtain an approximately normal distribution. Comparison of variables between groups was made using unpaired *t* test and chi‐square test. We examined the association between log‐transformed distensibility values and cardiovascular risk factors using unadjusted linear regression analysis and following adjustment for all other cardiovascular risk factors in multivariable regression analysis. Kaplan–Meier survival curves were plotted for outcome associated with high and low distensibility defined by the median distensibility value. The relationship between distensibility and time‐to cardiovascular event/death was investigated using univariable and multivariable Cox regression models. Subjects who were alive at the end of the follow‐up period without having experienced a cardiovascular event (May 31, 2020) were treated as right censored. Univariable Cox proportional hazards regression models were fitted for each time‐to‐death for log distensibility (Model 1). Multivariable Cox proportional hazards regression was performed using 2 models: first, adjusting for risk factors associated with distensibility in univariable regression analysis (where *P*<0.10, Model 2) and second, including all other cardiovascular risk factors. The analysis was repeated with PP and SI as the exposure variables. Kaplan–Meier survival curves were plotted for outcome associated with high and low PP and SI defined by the median values. All analysis was performed using STATA version 16 (StataCorp, College Station, TX) statistical software.

For quantitative assessment of the image segmentation network, we used the Dice metric. This evaluates the overlap between automated segmentation and the ground truth segmentation and has values between 0 and 100%, where 0 denotes no overlap and 100% perfect agreement. Bland–Altman analysis and Pearson's correlation coefficient were used to compare the systolic and diastolic areas obtained by the network with the ground truth values. For the QC network, sensitivity (% of manually labeled erroneous output that was correctly detected as “wrong” by the pipeline during QC), specificity (% of output manually labeled as error free that was not flagged by the pipeline during QC), and balanced accuracy were calculated.

## RESULTS

Participant characteristics are shown in Table 1. Mean (±SD) ages for men and women were 62.6±7.5 and 61.9±7.3 (*P*<0.0001) years, respectively. Average systolic and diastolic BP, height, weight, proportion of current smokers, treatment for hypertension and hypercholesterolemia, diabetes, and glucose were higher in men compared with women (each *P*<0.0001). Heart rate, total cholesterol, and HDL cholesterol were higher in women compared with men (each *P*<0.0001). Mean distensibility for men was lower compared with women at 1.77±1.15 and 2.10±1.45 10^−3^ mm Hg^−1^ (*P*<0.0001), respectively. Mean distensibility values were comparable to those in the MESA (Multi‐Ethnic Study of Atherosclerosis) study (overall mean aortic distensibility: 1.86±1.31).^6^, ^18^ Characteristics of participants excluded due to poor image quality are shown in Table S1–S4. Participants without distensibility data (n=3778) were older, had higher systolic and diastolic BP, total cholesterol, HDL‐cholesterol and were more likely to be on treatment for hypertension, hypercholesterolemia and diabetes compared with those with (n=8435) distensibility measures. These differences were broadly similar when comparing baseline characteristic in participants with distensibility to the total UK Biobank cohort (Table S2).

**Table 1:** Participant Characteristics in All Participants and by Sex

Characteristics	Total cohort (n=12 491)	Male participants (n=6492)	Female participants (n=5999)	*P* value
Age, y (n=12 491)	62.3±7.5	62.6±7.6	61.9±7.3	<0.0001
Systolic BP, mm Hg (n=12 213)	139.6±19.3	142.6±18.0	136.3±20.0	<0.0001
Diastolic BP, mm Hg (n=12 213)	79.6±10.6	81.6±10.3	77.4±10.5	<0.0001
Mean arterial pressure, mm Hg (n=12 213)	99.6±12.2	102.0±11.6	97.1±12.3	<0.0001
Heart rate, bpm (n=12 213)	68.2±11.6	66.9±12.0	69.3±11.1	<0.0001
Height, cm (n=12 491)	171.0±9.4	177.5±6.5	163.9±6.4	<0.0001
Weight, kg (n=12 491)	75.6±14.8	83.0±12.9	67.6±12.3	<0.0001
Current smoker, % (n=12 456)	458 (3.7)	277 (4.3)	181 (3.0)	<0.0001
Hypertension, % (n=12 343)	2167 (17.6)	1365 (21.3)	802 (13.5)	<0.0001
Hyperlipaemia, % (n=12 343)	2027 (22.3)	1430 (22.3)	597 (10.1)	<0.0001
Diabetes, % (n=12 466)	493 (4.0)	342 (5.3)	151 (2.5)	<0.0001
Cholesterol, mmol/L (n=11 795)	5.81 (1.1)	5.72 (1.0)	5.90 (1.1)	<0.0001
High‐density lipoprotein cholesterol, mmol/L (10 880)	1.50 (0.4)	1.34 (0.3)	1.68 (0.4)	<0.0001
Glucose, mmol/L (10 869)	5.01 (0.9)	5.04 (1.0)	4.97 (0.8)	<0.0001
Distensibility, 10^−3^ mm Hg^−1^(n=8435)	1.92±1.3	1.77±1.15	2.10±1.45	<0.0001

BP indicates blood pressure.

### Automatic Image Analysis

**Figure 1:**
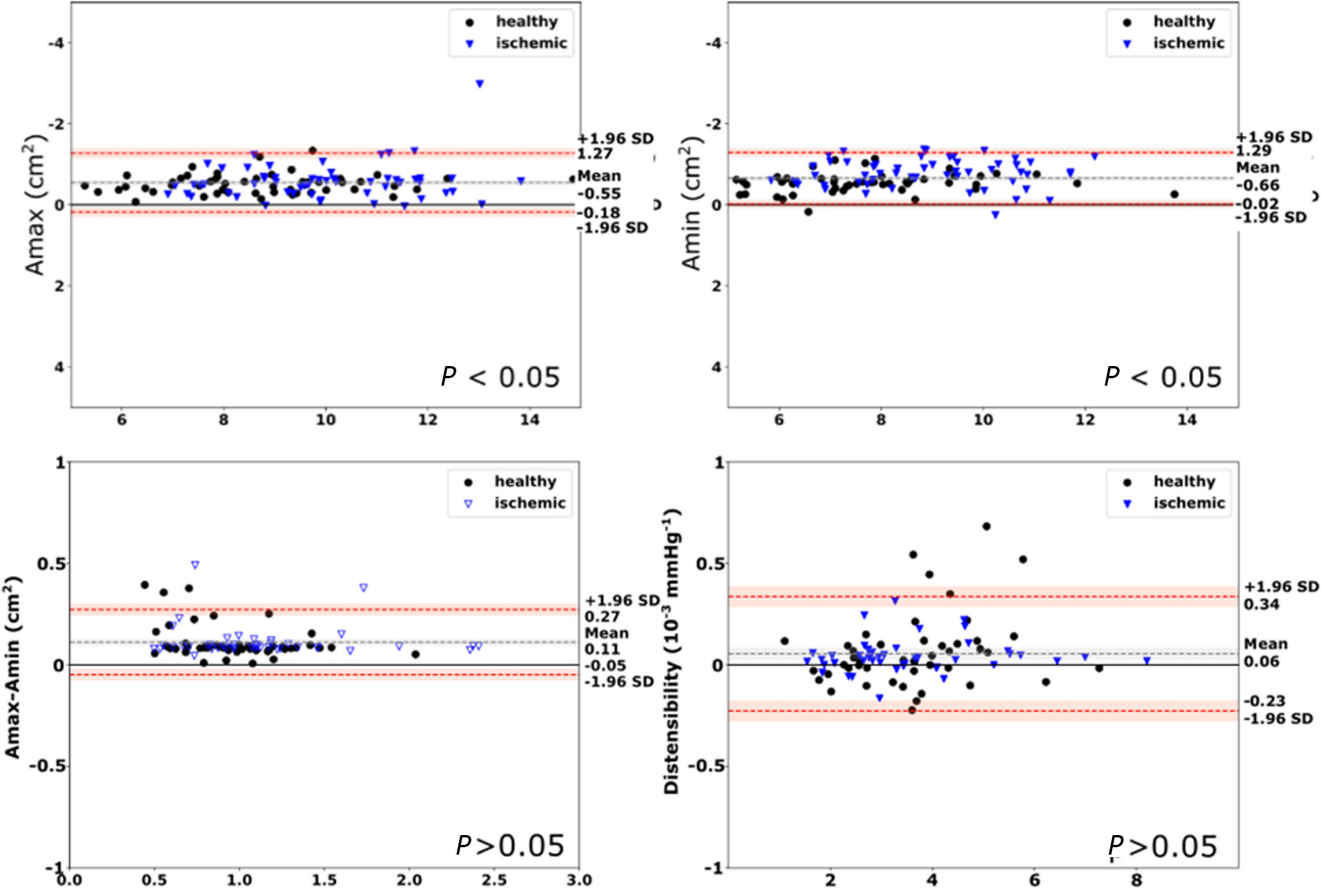
Bland–Altman plots for maximal and minimal aortic area (Amax and Amin, respectively), difference in area and distensibility for our automated analysis versus manual analysis.

Overall, the Dice score between manual and automated segmentations was 94.21±1.53% (93.87±1.01% for healthy and 94.57±1.84% for patients with cardiac disease respectively). There was a good correlation between automatically and manually obtained systolic and diastolic areas (Amin, *r*=0.980; Amax, *r*=0.985). Bland–Altman plots for agreement between the automated and manual analysis for areas, difference in areas and distensibility are shown in Figure 1. There was no significant bias for Amax, Amin or distensibility. For the QC network, sensitivity was 97.03%, specificity was 98.20%, and balanced accuracy was 97.60%. Based on a visual assessment, we found that the majority of rejections in our QC did not arise from technically unsatisfactory segmentation of the aortic area, but motion in the images and/or changes in real volumes that were likely the result of through plane motion of the aorta through the imaging plane.

### Distensibility and Cardiovascular Risk Factors

Table 2 shows the association between distensibility and cardiovascular risk factors. In unadjusted regression analysis, distensibility was negatively associated with age, MAP, heart rate, sex, total cholesterol, glucose, current smoking, hypertension, hypercholesterolemia and diabetes. In the fully adjusted model, age, MAP, heart rate, height, weight, glucose and hypertension remained negatively associated with aortic distensibility. There was no evidence of an association between aortic distensibility and sex, total cholesterol, HDL‐cholesterol, smoking status, or presence of diabetes.

**Table 2:** Univariable and Multivariable Linear Regression Analysis Between Distensibility and Cardiovascular Risk Factors

	Univariable regression			Multivariable regression		
	beta	95% CI	*P* value	beta	95% CI	*P* value
Age, y	−0.05	−0.052 to −0.048	<0.0001	−0.05	−0.047 to −0.043	<0.0001
Mean arterial pressure, mm Hg	−0.02	−0.021 to −0.019	<0.0001	−0.02	−0.02 to −0.01	<0.0001
Heart rate, bpm	−0.01	−0.008 to −0.006	<0.0001	−0.01	−0.006 to −0.004	<0.0001
Height, cm	0.00	−0.002 to 0.001	0.941	−0.01	−0.01 to 0.00	<0.0001
Weight, kg	−0.001	−0.002 to 0.001	0.114	0.004	0.003–0.005	<0.0001
Sex (female, male)	−0.13	−0.15 to 0.10	<0.0001	0.01	−0.02 to 0.05	0.508
Cholesterol, mmol/L	−0.07	−0.09 to −0.06	<0.0001	−0.01	−0.02 to 0.00	0.200
High‐density lipoprotein cholesterol (mmol/L)	0.01	−0.03 to 0.05	0.743	0.02	−0.01 to 0.06	0.199
Glucose (mmol/L)	−0.08	−0.10 to −0.06	<0.0001	−0.03	−0.05 to −0.02	<0.0001
Current smoker (no, yes)	0.16	0.09–0.23	<0.0001	−0.03	−0.09 to 0.03	0.326
Hypertension (no, yes)	−0.35	−0.39 to −0.32	<0.0001	−0.05	−0.08 to −0.01	<0.001
Hypercholesterolemia (no, yes)	−0.30	−0.34 to −0.27	<0.0001	0.02	−0.01 to 0.06	0.229
Diabetes (no, yes)	−0.19	−0.27 to −0.12	<0.0001	0.04	−0.03 to 0.11	0.226

### Pulse Pressure, Arterial Stiffness Index, and Cardiovascular Risk Factors

Mean PP and SI were 58.7+14.4 mm Hg and 9.52+2.8 m/sec, respectively. Table S3 shows the associations between PP and SI with cardiovascular risk factors. In the fully adjusted regression analysis, PP was positively associated with age, MAP, glucose, treatment for hypertension and diabetes and negatively with heart rate, weight and male sex. There was no significant association with height, total cholesterol, HDL‐cholesterol, current smoking and hypercholesterolemia. After adjustment, SI was positively associated with age, MAP, heart rate, height, weight, male sex, current smoking and treatment for hypertension. There was no significant association of SI with total cholesterol, HDL‐cholesterol, glucose, hypercholesterolemia and diabetes.

### Distensibility and New‐Onset Cardiovascular Events

**Figure 2:**
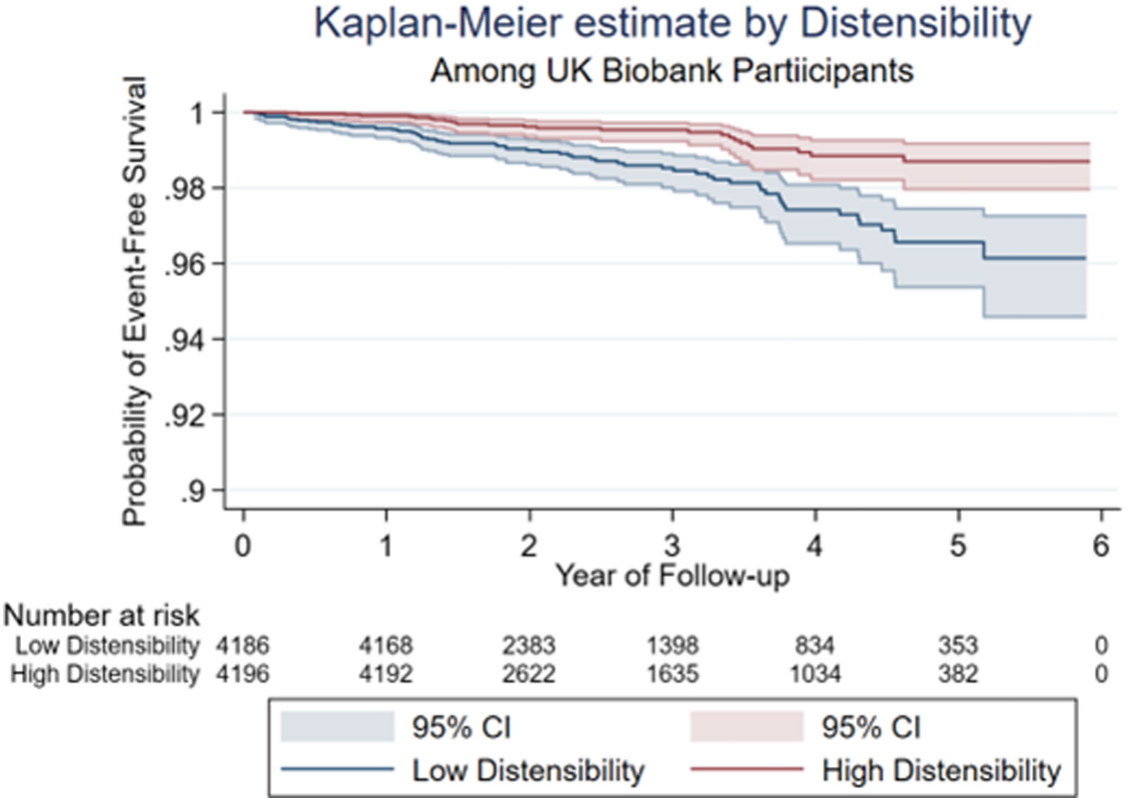
Kaplan–Meier curves showing probability of event‐free survival by distensibility.

Forty‐eight participants had experienced a cardiovascular event between the baseline and imaging visit and were excluded from further analysis and 4 participants were lost to follow‐up leaving 8382 included in subsequent analysis. Mean duration of follow‐up of participants was 2.8+1.3 years. Over the follow‐up period, 86 participants experienced a primary cardiovascular event of which 6 were cardiovascular death (cardiovascular event subtypes are outlined in Table S4). The total incidence rate for a cardiovascular event was 3.97 per 1000 person‐years. In participants with low distensibility versus high distensibility, incidence was 5.99 per 1000 person‐years compared with 2.08 per 1000 person‐years. Kaplan–Meier curves for probability of event‐free survival by high and low distensibility values are shown in Figure 2. Log‐rank testing confirmed a significant difference in event‐free survival between low and high distensibility groups, with low distensibility having a lower probability of event free survival compared with the high distensibility group (*P*<0.0001). Univariable Cox regression analysis showed that higher distensibility was associated with a lower risk of having a cardiovascular event (hazard ratio: 0.42 (95% CI: 0.33–0.55), per log‐unit change in distensibility, *P*<0.0001, Table 3). Adjustment for age, MAP, heart rate, height, weight, glucose and hypertension treatment, in multivariable Cox regression did not alter that association although it did modestly attenuate the estimate of lower hazard associated with higher distensibility (hazard ratio: 0.61 (95% CI: 0.41–0.91), *P*=0.016, Table 3). Further adjustment for other cardiovascular risk factors did not substantially change the association between distensibility and cardiovascular events. Distensibility is a direct local measure of aortic stiffness, calculated as the relative change in aortic area divided by the local aortic PP. The predictive value of diastolic aortic lumen area (in the denominator) was comparable to distensibility with a standardized hazard ratio of 1.35 (95% CI: 1.07–1.70) versus 0.73 (95% CI: 0.56–0.95) for distensibility.

**Table 3:** Relationship Between Aortic Distensibility With Incidence Cardiovascular Event

Cox regression model	Hazard ratio	95% CI	*P* value
Log distensibility			
Univariable	0.42	0.33–0.55	<0.0001
Multivariable*	0.61	0.41–0.91	0.016
Pulse pressure			
Univariable	1.02	1.01–1.04	<0.0001
Multivariable†	1	0.98–1.02	0.715
Arterial stiffness index			
Univariable	1.09	1.02–1.17	0.014
Multivariable‡	1.02	0.95–1.10	0.535

HR indicates heart rate; and MAP, mean arterial pressure.

* Adjusted for age, MAP, HR, hypertension, glucose, height, weight.

^†^ Adjusted for age, MAP, HR, hypertension, sex, weight, glucose, diabetes.

^‡^ Adjusted for age, MAP, HR, hypertension, sex, height, weight, current smoker.

### Pulse Pressure, Arterial Stiffness Index, and New‐Onset Cardiovascular Events

**Figure 3:**
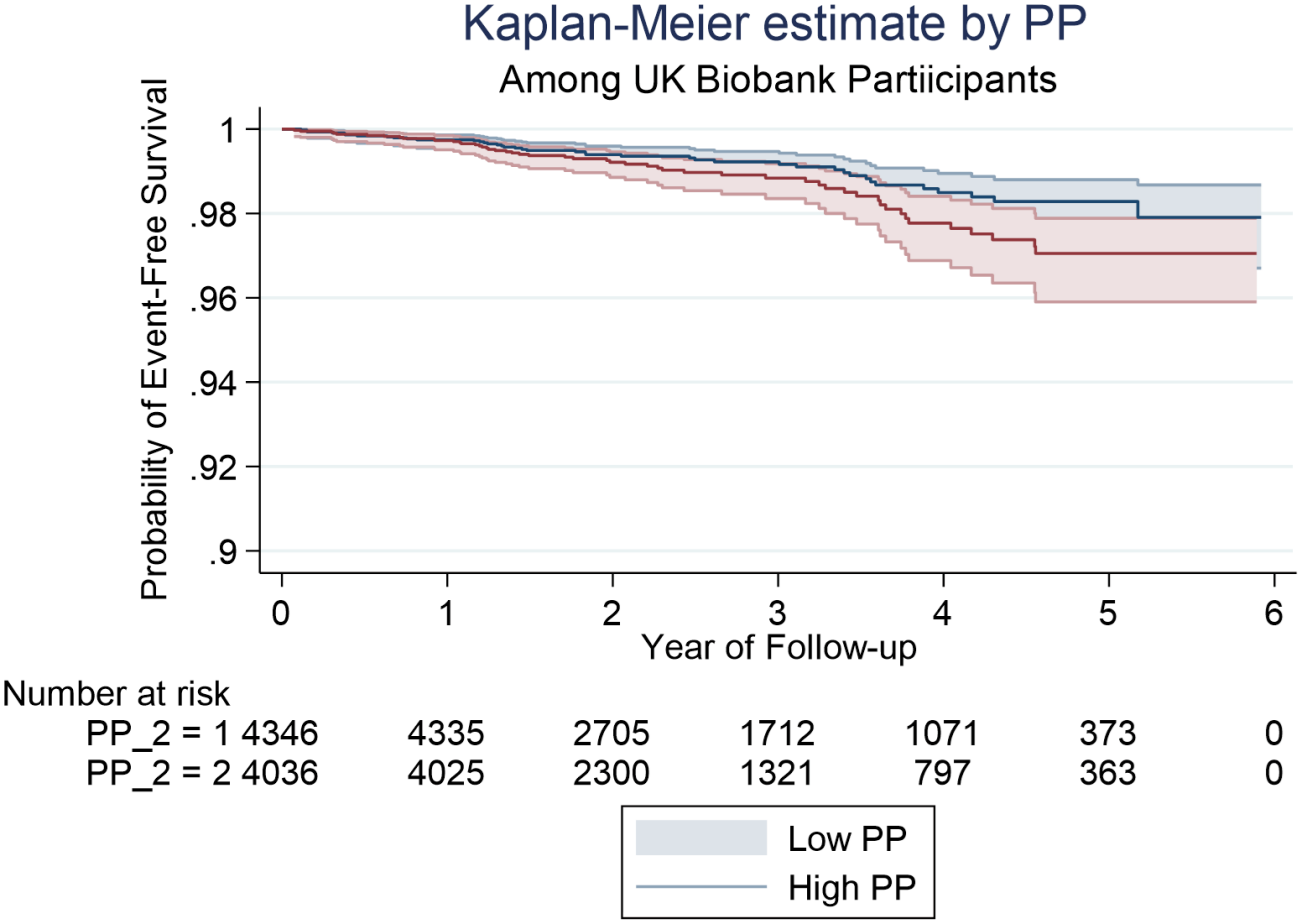
Kaplan–Meier curves showing probability of event‐free survival by pulse pressure (PP).

**Figure 4:**
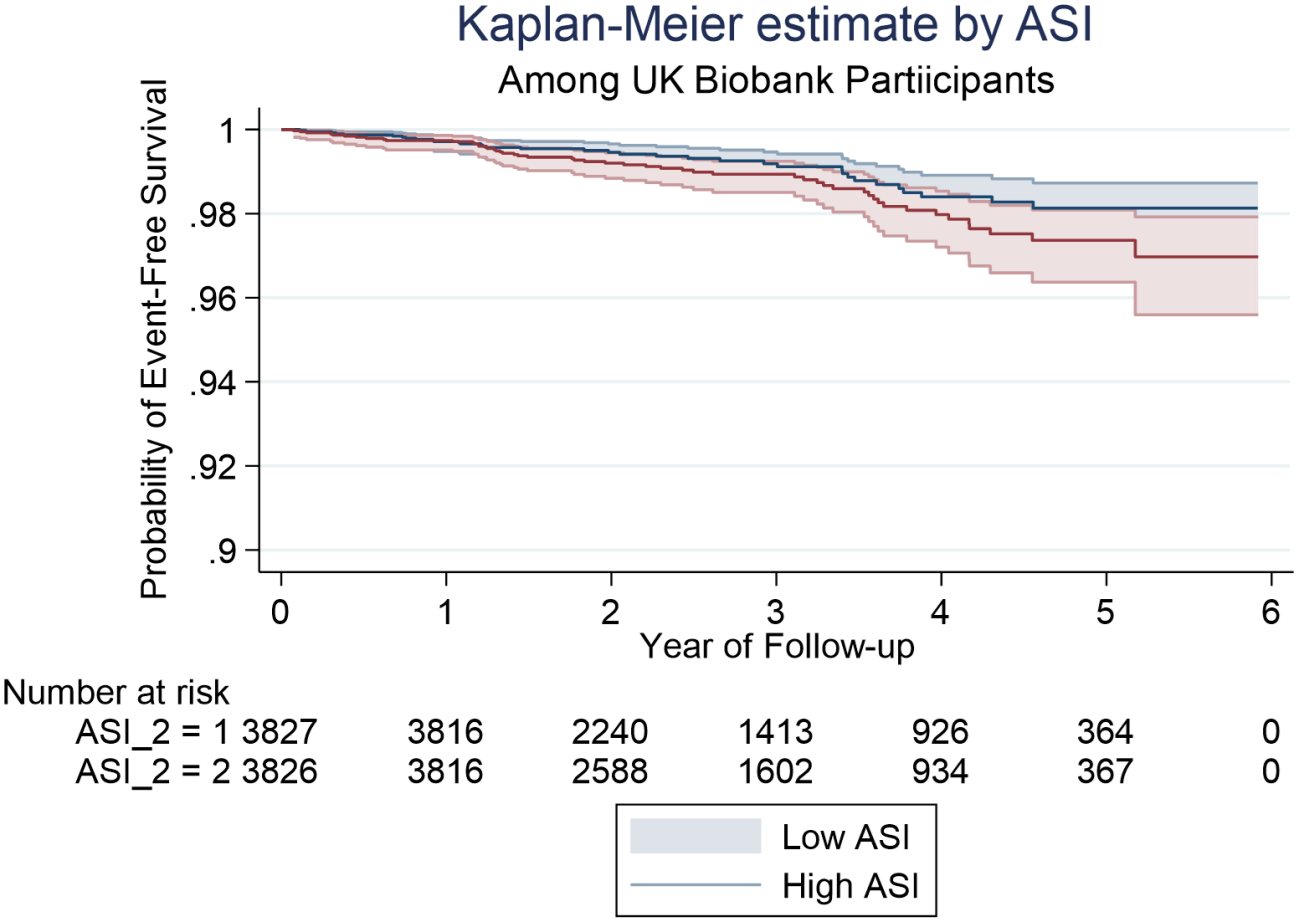
Kaplan–Meier curves showing probability of event‐free survival by arterial stiffness index (ASI).

In participants with low PP values (defined by median PP) the incidence rate of a cardiovascular event was 2.43 per 1000 person‐years compared with 3.60 per 1000 person‐years in participants categorized as having high PP. Kaplan–Meier curves for probability of event‐free survival by high and low PP are shown in Figure 3. Log‐rank testing suggested a difference in event‐free survival between low and high PP groups with individuals with high PP having a lower probability of event free survival (*P*=0.0672). Univariable Cox regression analysis showed that higher PP was associated with a higher risk of having a cardiovascular event (hazard ratio: 1.02 (95% CI: 1.01–1.04), *P*<0.0001, Table 3). However, after adjustment for age, MAP, heart rate, weight, glucose, diabetes and hypertension treatment in multivariable Cox regression, there was no significant association between PP and cardiovascular events (hazard ration: 1.00 (95% CI: 0.98–1.02), *P*=0.715, Table 3). Cardiovascular incidence rate by SI groups were 3.29 and 4.64 per 1000 person‐years for low versus high SI, respectively. Kaplan–Meier curves for probability of event‐free survival by high and low SI values are shown in Figure 4. There was no significant difference in event‐free survival between low and high SI groups on either univariable or multivariable Cox regression analysis (Table 3).

## DISCUSSION

To the best of our knowledge, this is the largest population study to examine aortic distensibility measured by automated CMR. We find that higher aortic distensibility is associated with reduced risk of cardiovascular event (adjusted hazard ratio, 0.61 [95% CI, 0.41–0.91]). In contrast, in the same individuals, we found no evidence of an association between the indirect measures of arterial stiffness, PP and SI, with adverse cardiovascular events. The finding of an association between aortic distensibility and cardiovascular events is in line with those of previous smaller studies. In 3675 participants of MESA, ascending aortic distensibility was a predictor of all‐cause mortality and cardiovascular events.^6^ In 2122 Dallas Heart Study participants, lower aortic distensibility was associated with higher risk of cardiovascular events.^7^

Previous studies investigating the association of the indirect measures of aortic stiffness PP and SI with cardiovascular events, after adjustment for other components of BP such as systolic BP, have reported inconsistent findings that may vary with age.^19^, ^20^ In 2232 Framingham Heart Study participants, central PP measured by arterial tonometry and brachial PP were not significant predictors of adverse cardiovascular events. In contrast, PWV, a more direct measure of arterial stiffness, was predictive of cardiovascular events.^21^ Similarly, in longitudinal follow‐up of 1126 participants in the PARTAGE (Predictive Values of Blood Pressure and Arterial Stiffness in Institutionalized Very Aged Population) study, PP was not independently predictive of cardiovascular events.^22^ In contrast, in 7336 hypertensive patients from the Campania Salute Network, higher PP was a predictor of major cardiovascular events.^23^ In other studies, PP was also predictive of cardiovascular events in patients on chronic hemodialysis,^24^ after myocardial infarction,^25^ and in elderly individuals.^26^ Findings from the 3520 Strong Heart Study participants suggest that central rather than peripheral PP may be a stronger predictor of future cardiovascular events.^27^ In a recent study in a larger UK Biobank cohort investigating the association between PP and SI with cardiovascular outcome, Said et al.^15^ found both PP and SI to associate with cardiovascular events in 169 613 participants. In the present study with just over 8000 participants, we did not find evidence of an association between PP or SI with cardiovascular events. The lack of association with PP and SI in the present study is likely to be explained by a smaller sample size compared with the findings of Said et al.^15^ and the variable findings in other studies explained by a modest association between PP and events that reaches significance only with large sample sizes. In addition, SI, which is derived from finger photoplesmography could reflect not only large artery stiffness but also various other vascular phenomena that affect hand microcirculation and may explain the difference in association.

Our present findings of an association of aortic distensibility but not PP or SI with cardiac events suggests that distensibility is a more sensitive measure of cardiovascular aging, more strongly associated to adverse cardiovascular outcome. Aortic distensibility is a direct measure of local aortic stiffness whereas PP and SI are influenced by other factors. PP, although related to aortic stiffness, is influenced by left ventricular contraction, aortic anatomy, and pressure wave reflection.^3^, ^4^ SI is derived from finger photoplethysmography to obtain a digital volume pulse that is characterized by 2 distinct waves. SI is measured as height divided by the time between the time between the peaks of the 2 waves on the assumption that this is related to the time of pressure propagation from central to peripheral sites and that height is a surrogate of path length. It has been shown to correlate modestly to PWV but also to be influenced by many other factors that determine peripheral pulse wave morphology.^9^, ^28^ The stronger association of distensibility with cardiovascular events than indirect measures of stiffness is consistent with the strong and universal association of carotid‐femoral PWV with cardiovascular events.^29^, ^30^ It suggests that distensibility can provide useful information on stiffness. As with carotid‐femoral PWV, it suggest that the prognostic impact of stiffness is greater than that of the PP generated by the arterial stiffening. This may be owing to direct measures of aortic stiffness providing a more complete characterization of the dynamic load on the left ventricle than BP, its impact on the microvasculature, and/or as a surrogate of other aspects of aging.

We also investigated the association between aortic distensibility, PP, and SI with conventional cardiovascular risk factors. Aortic distensibility associated with age, MAP, heart rate, and glucose but not with other conventional cardiovascular risk factors such as cholesterol, smoking, and male sex. This is consistent with previous studies where regional aortic stiffness, measured as PWV, strongly relates to age and BP but not to other risk factors associated with atherosclerosis.^31^

**Figure 5:**
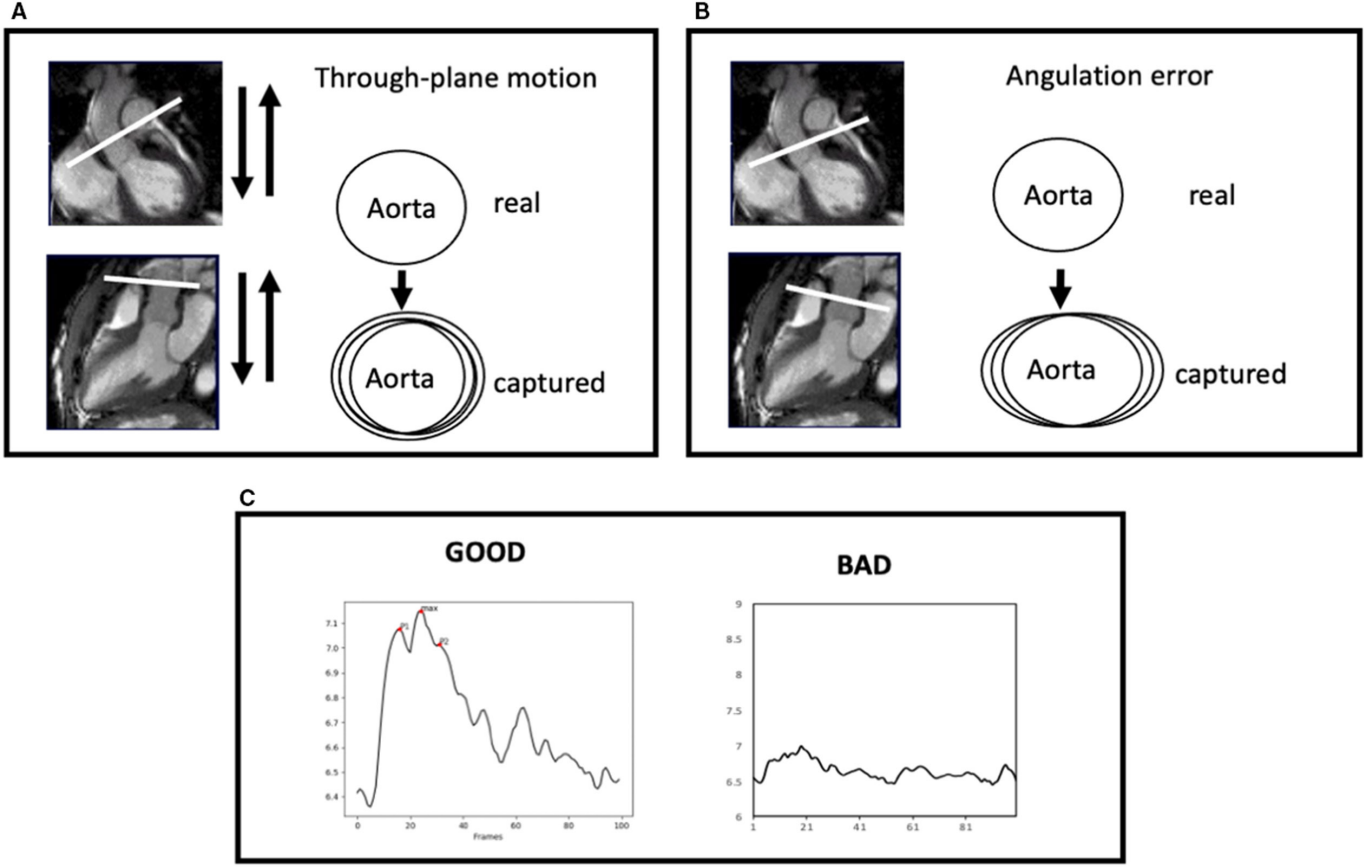
Example of aortic distensibility image analysis from UK Biobank participant. Aortic segmentation and impact of through plane motion on aortic areas (**A**) and slight angulations in planning on aortic segmentations (**B**). Through plane motion and angulated acquisitions result in additional changes in aortic area curves not directly related to the pressure dynamics over the cardiac cycle. Our quality control algorithm interrogates the aortic area curves for expected to detect erroneous curves with unphysiological behavior, for example the complete absence of systolic area increase (**C**). Reproduced by kind permission of UK Biobank ©.

To obtain the aortic distensibility biomarkers, we used a deep learning image segmentation framework. Deep learning provides human‐level accuracy in a variety of medical image‐segmentation tasks. A drawback is its “black box” design, which makes it challenging to anticipate and prevent potential errors in analysis. Applying deep learning algorithms without robust QC is therefore undesirable. In our framework, we integrate an additional QC step, which takes into account the known physiology of aortic dimensional changes over the cardiac cycle to screen for potential errors, resulting in an accurate error detection within the automated method. We have previously applied this QC successfully in segmenting left ventricular short‐ and long axis cine CMR. This approach was particularly relevant to the specific task of aortic cine segmentation. Minor planning errors or breathing motion during acquisitions can result in an adaption of the aortic dimensions that do not arise from the cardiac cycle itself. Indeed, we found that in a significant proportion of cases such acquisition errors resulted in erroneous distensibility curves, making a QC step vital for reporting accurate findings. Of note, the majority of QC issues did not arise from technically unsatisfactory images or segmentation of the aortic area, but motion in the images and/or unrealistic changes in aortic areas (that were likely the result of through‐plane motion of the aorta through the imaging plane (see examples in Figure 5)).

Although advocated in clinical practice for risk stratification (and with other potential clinical applications), measurement of aortic stiffness has not been performed in clinical practice because of the practical difficulties in performing measurements like carotid‐femoral PWV.^32^ The present study shows that the automated CMR has promise as a measurement that can be incorporated, with minimal additional overhead, into routine clinical imaging. Although the use of CMR as the preferred imaging modality in assessment of cardiovascular disease varies according to local resources, the development of low‐field MRI is likely to make it more widely available and the incorporation of the additional risk stratification provided through measurement of automated CMR‐derived aortic distensibility could have considerable clinical utility.

### Strengths and Limitations

To the best of our knowledge, this is the largest study to investigate the association between aortic distensibility, with adverse cardiovascular outcome. This study is limited by a relatively short follow‐up duration, with relatively few cardiovascular events occurring over this time. However, even during this period we were able to show the sensitivity of aortic distensibility to predict risk of cardiovascular events. Unfortunately, because of this we were not able to consider the association with subtypes of cardiovascular events. PP measured in the brachial artery is an overestimation of PP at the site of aortic diameter measurements owing to peripheral amplification of the pressure pulse as it travels from central to peripheral sites. However, amplification is less marked in middle=aged to older individuals ^33^ in as in this study in whom risk assessment is most relevant. Furthermore, noninvasive technology that seeks to estimate aortic PP from peripheral PP is affected by inaccuracies in the true measurement of intra‐arterial pressure.^34^ Aortic distensibility measurements employ PP for calculation and are therefore subject to the same limitation. Aortic distensibility measures are determined by diastolic lumen area (which appears in the denominator of the expression for distensibility) and we found a predictive effect of lumen area as well as distensibility. However, this may be in part because of the reduced experimental error in measuring lumen area relative to distensibility. Further studies will be required to determine the relative importance of lumen area compared with distensibility. We were not able to measure aortic distensibility in >30% of individuals because of motion artifact and cannot exclude the possibility that this may have affected our point estimates of distensibility. To compute risk associated with reduced distensibility on an individual basis, further work is required to both reduce motion artifact and to make the algorithm more tolerant to motion artifact. Quantification of both axial and circumferential strain could further refine the measurement of distensibility but whether this would increase its prognostic power would need to be tested.

## CONCLUSIONS

Aortic distensibility determined by automated CMR analysis, but not PP or SI, significantly predicted new‐onset cardiovascular events in over 8000 participants from UK Biobank. Aortic distensibility by automated CMR is a promising tool to examine vascular aging and may overcome the practical limitations of more classical measures of aortic stiffness that have prevented their widespread use.

## Disclosures

None.

## Sources of Funding

The authors acknowledge financial support the National Institute for Health Research (NIHR) Cardiovascular MedTech Co‐operative (previously existing as the Cardiovascular Healthcare Technology Co‐operative 2012–2017) award to the Guy's and St Thomas' National Health Service (NHS) Foundation Trust, in partnership with King's College London and the NIHR comprehensive Biomedical Research Centre of the Guy's & St Thomas' NHS Foundation Trust. This work was also supported by the Wellcome/EPSRC Centre for Medical Engineering at Kings College London (WT 203148/Z/16/Z). The views expressed are those of the author(s) and not necessarily those of the NHS, the NIHR, Wellcome Trust, EPSRC, or the Department of Health. This research was funded in part by the Wellcome Trust for the purpose of open access, the author has applied a CC BY public copyright licence to any Author Accepted Manuscript version arising from this submission.

## Supporting information

Tables S1–S4
