# Supplementary material for: Aortic Distensibility Measured by Automated Analysis of Magnetic Resonance Imaging Predicts Adverse Cardiovascular Events in UK Biobank
Source: J Am Heart Assoc. 2022 Dec 6;11(23):e026361. doi: 10.1161/JAHA.122.026361 (PMC9851433; doi:10.1161/JAHA.122.026361)
Supplement: Supplementary file 1 — Tables S1–S4 [file JAH3-11-e026361-s001.pdf]

Supplemental Material

Table S1. Participant characteristics in those with and without distensibility analysis.

Characteristics	Total Cohort (n=12,491)	Without distensibility (n=4,056)	With distensibility (n=8,435)	P-value
**Age, years (n=12,491)**	62.3 ± 7.5	64.5 ± 7.2	61.2 ± 7.4	<0.001
**SBP, mmHg (n=12,213)**	139.6 ± 19.3	142.8 ± 19.7	138.1 ± 18.9	<0.001
**DBP, mmHg(n=12,213)**	79.6+10.6	80.1+ 10.6	79.4 ± 10.6	<0.001
**MAP, mmHg (n=12,213)**	99.6+12.2	101.0+12.2	99.0 + 12.1	<0.001
**Heart rate, bpm (n=12,213)**	68.2+11.6	69.6+12.1	67.6 + 11.4	<0.001
**Height, cm (n=12,491)**	171.0+9.4	170.1 + 9.3	171.4 + 9.4	<0.001
**Weight, kg (n=12,491)**	75.6+14.8	75.1 + 15.4	75.8 + 14.5	<0.01
**Current Smoker, % (n=12,456)**	458 (3.7)	147 (3.6)	311 (3.7)	0.851
**Hypertension, % (n=12,343)**	2,167 (17.6)	832 (20.8)	1,335 (16.0)	<0.001
**Hypercholesterolemia, % (n=12,343)**	2,027 (22.3)	781 (19.5)	1,246 (14.9)	<0.001
**Diabetes mellitus, % (n=12,466)**	493 (4.0)	184 (4.6)	309 (3.7)	<0.05
**Cholesterol, mmol/L (n=11,795)**	5.81 (1.1)	5.84 (1.1)	5.80 (1.0)	<0.05
**HDL Cholesterol, mmol/L (10,880)**	1.50 (0.4)	1.53 (0.4)	1.49 (0.4)	<0.001
**Glucose, mmol/L (10,869)**	5.01 (0.9)	5.03 (0.9)	4.99 (0.9)	0.0845

SBP= systolic blood pressure, DBP – diastolic blood pressure; MAP=mean arterial pressure, HDL=high-density lipoprotein.

Table S2. Participant baseline visit characteristics in those with distensibility measurements and total UK Biobank cohort.

**Characteristics**	**Total Cohort (n=494,069)**	**With distensibility (n=8,435)**	**P-value**
**Age, years**	56.6 ± 8.1	52.8 ± 7.3	<0.001
**SBP, mmHg**	137.7 ± 26.6	104.4 ± 62.2	<0.001
**DBP, mmHg**	80.9 ±15.1	65.3 ± 35.1	<0.001
**Heart rate, bpm**	69.6 ±11.7	66.9 ± 10.6	<0.001
**Weight, kg**	78.1 ± 16.0	76.4 ± 14.3	<0.001
**Current Smoker, %**	52,452 (10.6)	526 (6.2)	<0.001
**Cholesterol, mmol/L**	5.69 ± 1.1	5.74 ± 1.0	<0.001
**HDL Cholesterol, mmol/L**	1.45 ± 0.4	1.47 ± 0.4	<0.001
**Glucose, mmol/L**	5.13 ± 1.2	4.96 ± 0.9	<0.001

SBP= systolic blood pressure, DBP – diastolic blood pressure; MAP=mean arterial pressure, HDL=high-density lipoprotein.

Table S3. Multivariable linear regression analysis between pulse pressure and arterial stiffness index with cardiovascular risk factors.

	Pulse pressure		Arterial stiffness index	
	**Beta (SE)**	**P-Value**	**Beta (SE)**	**P-Value**
Age, (years)	0.58 (0.02)	<0.0001	0.03 (0.01)	<0.0001
MAP, (mm Hg)	0.57 (0.01)	<0.0001	0.02 (0.003)	<0.0001
HR, (bpm)	-0.19 (0.01)	<0.0001	0.03 (0.003)	<0.0001
Height, (cm)	0.003 (0.02)	0.17	0.02 (0.01)	<0.01
Weight, (kg)	-0.07 (0.01)	<0.0001	0.01 (0.003)	<0.0001
Gender	-0.77 (0.43)	0.072	0.43 (0.11)	<0.0001
Cholesterol, mmol/L	0.17 (0.14)	0.211	0.01 (0.03)	0.783
HDL-Cholesterol, mmol/L	-0.47 (-.43)	0.282	-0.16 (0.11)	0.783
Glucose, mmol/L	0.77 (0.17)	<0.0001	0.03 (0.04)	0.478
Current smoker	0.31 (0.72)	0.669	0.65 (0.18)	<0.0001
Hypertension	0.95 (0.41)	0.020	-0.21 (0.10)	<0.05
Hypercholesterolemia	0.18 (0.43)	0.673	0.14 (0.11)	0.213
Diabetes mellitus	3.07 (0.83)	<0.0001	0.05 (0.21)	0.798

Table S4. Cardiovascular event subtypes.

ICD10 Code	Number of Events/Cause of death
I10 Essential (primary) hypertension	1
I20.9 Angina pectoris, unspecified	3
I21.0 Acute transmural myocardial infarction of anterior wall	3
I21.1 Acute transmural myocardial infarction of inferior wall	1
I21.2 Acute transmural myocardial infarction of other sites	1
I21.4 Acute subendocardial myocardial infarction	8
I21.9 Acute myocardial infarction, unspecified	1
I24.9 Acute ischaemic heart disease, unspecified	3
I25.1 Atherosclerotic heart disease	24
I25.4 Coronary artery aneurysm	1
I25.9 Chronic ischaemic heart disease, unspecified	1
I35.0 Aortic (valve) stenosis	1
I48 Atrial fibrillation and flutter	1
I48.0 Paroxysmal atrial fibrillation	2
I49.9 Cardiac arrhythmia, unspecified	1
I60.2 Subarachnoid haemorrhage from anterior communicating artery	1
I60.9 Subarachnoid haemorrhage, unspecified	1
I61.1 Intracerebral haemorrhage in hemisphere, cortical	1
I61.3 Intracerebral haemorrhage in brain stem	1
I61.9 Intracerebral haemorrhage, unspecified	1
I63.3 Cerebral infarction due to thrombosis of cerebral arteries	8
I63.5 Cerebral infarction due to unspecified occlusion or stenosis of	4
cerebral arteries	
I63.9 Cerebral infarction, unspecified	17
